# Efficacy assessment of antiretroviral drugs against equine infectious anemia virus *in vitro*

**DOI:** 10.1016/j.virusres.2024.199503

**Published:** 2024-12-11

**Authors:** Cécile Schimmich, Astrid Vabret, José-Carlos Valle-Casuso

**Affiliations:** a ANSES Animal Health Laboratory, PhEED Unit, Goustranville, France; b University of Caen Normandy, Dynamicure INSERM UMR 1311, Centre hospital-universitaire (CHU) Caen, Department of Virology, Caen, France; c Mixed technological Unit “Equine Health and Welfare – Organisation and Traceability of the Equine Industry” (UMT SABOT), France

**Keywords:** EIAV, HIV-1, Antiviral drugs, Antiretroviral drugs, Lentivirus, Veterinary medicine

## Abstract

Equine infectious anemia virus (EIAV) is an equine *lentivirus* related to human immunodeficiency virus type 1 (HIV-1). Both viruses are related among the *Retroviridae* family, but their clinical manifestations are different as EIAV causes a long persistent infection with no progressive immune dysfunction in most cases. Today, no treatment is approved against EIAV, contrary to HIV-1, manageable through antiretroviral therapy, known as HAART (highly active antiretroviral therapy) or cART (combination antiretroviral therapy). No information about the efficacy of antiretroviral drugs against EIAV is available in the literature. This study evaluates the *in vitro* antiviral effect of eighteen FDA-approved antiretroviral compounds from different drug families, in an equine cells *in vitro* infection model with EIAV reference strain. Equine dermal cells, as well as equine peripheral blood mononuclear cells were treated with non-cytotoxic drug concentrations and infected with EIAV. Relative virus release in culture supernatants was assessed through relative quantification of viral RNA via RTqPCR and viral DNA comprising proviral integration in the cell genome was assessed through qPCR of infected cells, both after nucleic acid extractions. Out of eighteen tested drugs, thirteen showed a significant antiviral effect against EIAV *in vitro*, an interesting discovery showing the similarities between HIV-1 and EIAV and opening a possibility to treat equine infectious anemia to avoid the disease spread.

## Highlights

• Targeted screening of antiretroviral compounds anti-HIV-1 against EIAV *in vitro* infection of equine cells (ED cells and ePBMCs).
• 13 compounds were identified as effective against EIAV *in vitro*.
• Tested NRTIs, PIs and INSTIs have an antiviral effect against EIAV *in vitro* in equine cells.
• Tested NNRTIs, FI and CI have no antiviral effect against EIAV *in vitro* in equine cells.

## 1 Introduction

**Fig. 1:**
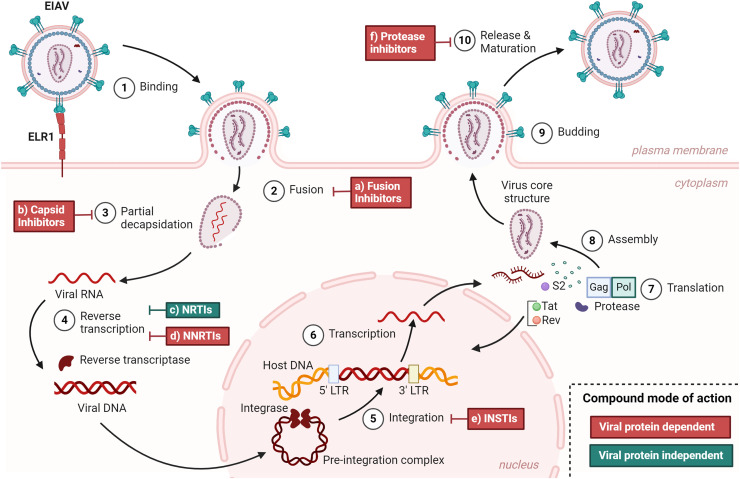
EIAV viral cycle and possible targets of antiretroviral compounds 1) Binding of the virus through the env protein, to the ELR1 receptor 2) Fusion of the viral membrane with the plasma membrane 3) Partial decapsidation - the viral capsid needs to be shed to allow the reverse transcription of the viral RNA into DNA 4) The viral reverse transcriptase allows reverse transcription of the viral RNA into the viral DNA 5) Integration of the viral DNA as the pre-integration complex (PIC) into the cellular DNA is mediated by the viral integrase and host factors inside the nucleus 6) Viral RNAs get transcribed by the cellular machinery 7) Viral proteins get translated from viral mRNA thanks to the cellular machinery 8) Assembly of new virions occurs in the cytoplasm, encapsidating viral RNA genomes 9) New virions are formed by the budding at the plasma membrane, allowing the virions to acquire their lipidic envelop 10) Virions get released from the plasma membrane and the viral protease allows the maturation of the viral proteins and the newly formed virions become infectious // a) Fusion inhibitors (FI) prevent fusion between the viral membrane and plasma membrane by interacting with HIV-1 envelop protein gp41 b) Capsid inhibitors (CI) interact with the capsid protein and prevent the uncoating of the viral RNA, preventing the import to the nuclear pore and the reverse transcription as well as reassembly of the capsid before virion release c) Nucleoside reverse transcriptase inhibitors (NRTI) are nucleoside analogues, with greater affinity for viral polymerases than cellular polymerases and that impair reverse transcription d) Non-nucleoside reverse transcriptase inhibitors (NNRTIs) impair the viral reverse transcriptase by binding to the enzyme directly e) Integrase strand transfer inhibitors (INSTIs) prevent the viral integrase to catalyze the integration reaction that allows viral DNA to integrate into the host DNA f) Protease inhibitors (PI) target the viral protease protein and prevent the maturation of viral proteins // Compound mode of action: Direct: the compound binds directly to the viral protein, impairing its antiviral action; Indirect: the compound has an antiviral effect without binding to the viral protein directly.

Equine infectious anemia virus (EIAV), discovered in 1904 (Vallée and Carré 1904), is the causative agent of equine infectious anemia, a disease affecting equids worldwide, transmitted by insect vectors (Hawkins et al., 1976). Equine infectious anemia is a dynamic disease characterized by three distinct phases (Leroux, Cadoré, and Montelaro 2004). To this date, there is no therapeutic alternative to treat infected equids and varying from country to country, mandatory euthanasia or isolation of positive tested equids can be the only option to manage the disease (Issel et al. 2014). EIAV is a *lentivirus* from the *Retroviridae* family, related to the human immunodeficiency virus (HIV), discovered in 1983 (Barré-Sinoussi et al. 1983) and causing the acquired immunodeficiency syndrome (AIDS) world pandemic, with 39 million people living with HIV in 2022 according to WHO (WHO 2022). As both lentiviruses, their viral cycles are very similar, and we can describe the EIAV cycle as follows (Fig. 1), similar to the HIV cycle (Fanales-Belasio et al. 2010). EIAV enters target equine cells via a single receptor, the equine *lentivirus* receptor 1 (ELR1) (Zhang et al. 2005) and contrary to HIV-1 (Clapham and McKnight 2002) and other lentiviruses such as simian immunodeficiency virus (SIV) and feline immunodeficiency virus (FIV) (De Parseval et al. 2004), no co-receptor have been described. After binding to ELR1, the fusion of viral envelop to the cell membrane occurs and the viral capsid enters the cytoplasm. The next step is reverse transcription of the viral RNA into the viral DNA. This is a key step in lentiviral infections, which is done by the viral reverse transcriptase. Then proviral DNA associated with host factors and the viral integrase as the pre-integration complex (PIC) is integrated into a host chromosome inside the nucleus. From this integrated provirus, copies of the viral genome can be transcribed, as well as mRNA to form the new viral proteins, translated in the cell cytoplasm. Finally, newly formed virions are matured thanks to the viral protease, allowing the release of infectious virions, after budding at the cell plasma membrane.

The understanding of the viral cycle was a key point in developing efficient anti-HIV-1 therapies, as all the developed drugs target specific steps of this cycle (Fig. 1). Highly active antiretroviral therapy (HAART) or combination antiretroviral therapy (cART) are the regimes of antiretroviral drugs used to manage HIV-1 infection, turning it into a manageable chronic illness.

Over thirty drugs have now shown effect against HIV-1, developed over the past 30 years (Shin, Park, and Yoon 2021). These drugs are classified by their mechanism of action and specifically at which viral step they counteract the infection (Fig. 1). There are nucleoside reverse transcriptase inhibitors (NRTIs) (Langtry and Campoli-Richards 1989) and non-nucleoside reverse transcriptase inhibitors (NNRTIs) (Namasivayam et al. 2019), both classes targeting the viral enzyme reverse transcriptase. Still acting in the early steps of the viral cycle, integrase strand transfer inhibitors (INSTIs) that block the integration of the provirus into the host cell genome (Engelman 2019), fusion inhibitors (FI) prevent fusion of the virus to the target cell (Wild et al. 1994). Later in the cycle, protease inhibitors (PIs) impair the maturation of viral precursor proteins (Palella et al. 1998). Lastly approved class are capsid inhibitors (CI) that not only impair uncoating at early stages of the infection but also prevent the encapsidation of newly synthetized viral genome (Di Perri 2023)**.**

While these compounds were shown to be active against HIV-1, at different step of the viral cycle, little is known about their efficacy against EIAV. The effect of several antiretroviral drugs was tested against on FIV (Hartmann, Wooding, and Bergmann 2015), a very studied non-primate *lentivirus*, that has similarities with HIV-1 at both molecular and clinical levels (Elder et al. 2008).

**Fig. 2:**
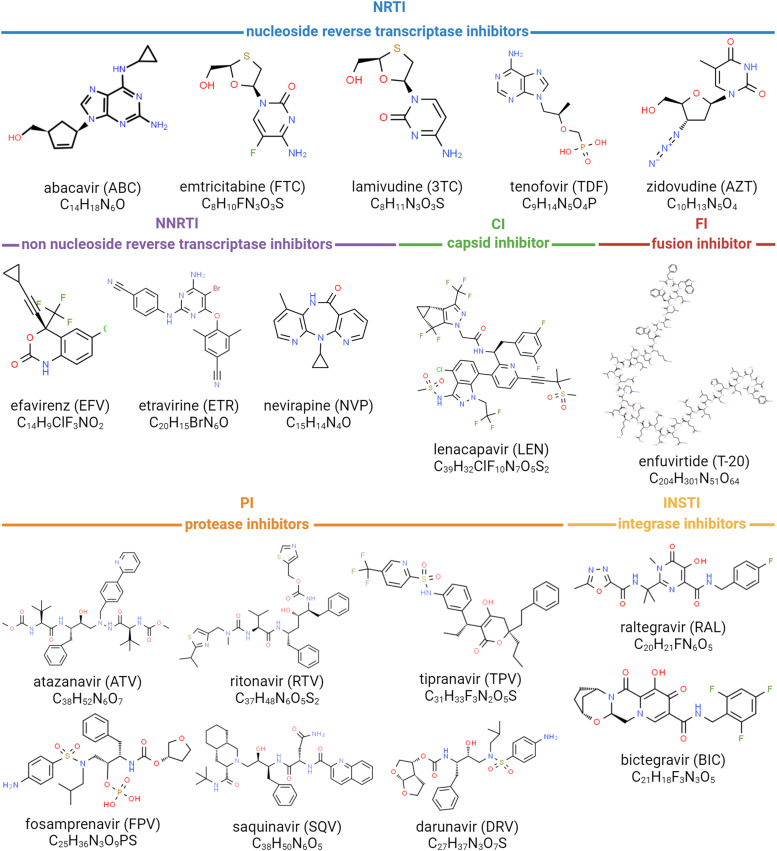
Molecular formulas of FDA-approved anti-HIV-1 compounds used in this study, FDA: Food and Drug Administration.

In this study, we show the efficacy of thirteen out of eighteen FDA-approved (Food and Drug Administration, USA) HIV-1 antiretroviral drugs, chosen to represent NRTIs, NNRTIs, PIs, INSTIs, FI, and CI (Fig. 2), against EIAV in an *in vitro* equine cell model. First, we evaluated the cytotoxicity of these compounds newly used on equine cells. Then, we tested their impact on viral release in culture supernatants and on viral DNA (integrated as a provirus or not) directly in infected equine dermal cells (ED cells) and primary equine peripheral blood mononuclear cells (ePBMCs).

## 2 Materials and methods

### 2.1 Compounds

Anti-HIV compounds tested in this study are the following: abacavir (ABC), atazanavir (ATZ), bictegravir (BIC), efavirenz (EFV), darunavir (DRV), emtricitabine (FTC), enfuvirtide (ENF), etravirine (ETR), fosamprenavir (FPV), lamivudine (3TC), lenacapavir (LEN), nevirapine (NVP), raltegravir (RAL), ritonavir (RTV), saquinavir (SQV), tenofovir disoproxil fumarate (tenofovir, TDF), tipranavir (TPV) and zidovudine (AZT), all purchased from Sigma-Aldrich (Sigma-Aldrich, St. Louis, MO, USA). Abacavir, lamivudine, and emtricitabine were dissolved in H_2_0 while all other compounds were dissolved in dimethyl sulfoxide (DMSO) (Sigma-Aldrich, St. Louis, MO, USA). Compounds were tested at final concentrations ranging from 0.1 to 300 µM for cytotoxic assay and from 0.1 µM to 200 µM for antiviral assays (supplementary material table S1), at maximum 1 % DMSO and compared to control condition without any drug but 1 % DMSO in required conditions.

### 2.2 Cell line culture

Equine dermal cells (ED cells) (E. derm NBM-6, ATCC CCL-57, Manassas, VA, USA) were maintained at 37 °C and 5 % CO_2_ in complete high glucose Dulbecco's Modified Eagle Medium (DMEM) (Gibco, Grand Island, NY, USA) supplemented with 1 mM of sodium pyruvate (Gibco), 50 U/ml of penicillin and 50 µg/ml of streptomycin (Gibco), 10 % heat inactivated fetal bovine serum (FBS) (Cytiva, Logan, UT, United States) and 0.025 M of HEPES (Gibco).

### 2.3 PBMC isolation and culture

Blood from healthy EIAV-negative horses was collected by a vet using citrate EDTA tubes. Equine peripheral blood mononuclear cells (ePBMCs) were isolated from this blood with Ficoll-Paque Plus (Cytiva). Briefly, blood was diluted to half with PBS, 30 ml of diluted blood was layered on top of 15 ml of Ficoll, centrifugation was conducted at 700 x *g* for 20 min with slow acceleration and deceleration at room temperature and ePBMCs were collected in the cloudy cell ring in the interphase. Collected ePBMCs were washed once with 45 ml of Roswell Park Memorial Institute 1640 (RPMI 1640, (Gibco)), centrifuged 10 min at 750 x g washed a second time in RPMI 3 % FBS with a 10 min, 700 x g centrifugation before counting the cell concentration.

Equine PBMCs were maintained in RPMI 1640 (Gibco) supplemented with 10 % FBS, 50U/ml penicillin 50µg/ml streptomycin, counted, seeded in required plates and maintained at 37 °C, 5 % CO_2_ incubator.

### 2.4 Virus preparation

ED cells were chronically infected with EIAV Wyoming strain (ATCC VR-778) directly and maintained in complete DMEM at 37 °C, CO_2_, the same manner as non-infected cells. When cells reached confluency, they were subcultured in the ratio of 1:3. No cytopathic effects were observed. When cells stopped growing for a few days, reaching a confluency plateau, new non-infected ED cells were added to boost growth. Virus containing culture supernatant was recovered every 72 h with a medium change for the cells. All supernatants recovered were frozen at −20 °C. They were checked for EIAV with RTqPCR, then pooled, filtered through 0.045 µm filters, and frozen in single-use aliquots. This viral stock was also titrated by detecting the virion associated reverse transcriptase activity using a reverse transcriptase assay (Roche Diagnostics, Rotkreuz, Switzerland), according to the manufacturer's instructions. The resulting value was 8ng.ml^-1^ of RT.

### 2.5 Infection of ED cells and ePBMCs

ED cells were harvested at 80 % confluency using 0.25 % Trypsin-EDTA and counted. After centrifugation, cells were resuspended in complete DMEM and were seeded in 24-well plates at 1.10^5^ cells per well. Viral stock was added at 100 µl per well for infected conditions. Compounds were added according to the tested condition. Infection was conducted for 24 h at 37 °C, 5 % CO_2_ in 400 µl final volume complete DMEM, then supernatant was removed, cells were washed 3 times in PBS and DMEM was added to every well, with or without additional molecule according to the determined test condition. Viral production or viral DNA was determined at 24, 72 or 144 h after this washing step. ePBMCS were seeded at 2.5.10^4^ cells per well in 96-well round bottom plates. Viral stock was added at 25 µl per well for infected conditions. Compounds were added according to the tested condition. Infection was conducted for 24 h at 37 °C, 5 % CO_2_ in 200 µl final volume complete RPMI, then plates were centrifuged at 220 x g for 10 min. Virus containing medium was removed and cells were washed three times in PBS (with centrifugation step before removing supernatant each time) and complete RPMI was added to every well, with or without additional molecule according to the determined test condition. Viral production or viral DNA was determined at 24, 72 or 144 h after this washing step.

### 2.6 Cytotoxicity assay

Cell viability was assessed at 1, 24, 72 and 144, hours after plating with tested compounds by quantification of cellular adenosine triphosphate (ATP) in 96-well black clear bottom plates (Greiner Bio-one, Kremsmünster, Austria) using the CellTiter-Glo (CTG) Luminescent Cell Viability Assay (Promega, Madison, WI, USA). For each molecule assay, displayed values correspond to the mean of three biological replicates composed of four technical replicates or eight for control conditions (no drug, 1 % DMSO).

ED cells or ePBMCs were seeded in 96-well black plates, clear bottom wells (Greiner Bio-one), 2.5.10^4^ cells per well, cultured with respective complete culture medium. A concentration range was determined by literature study and seven to ten concentrations were tested. After 1, 24, 72 or 144 h, corresponding plates were retrieved from the incubator. Culture medium was removed and 100 µl of room temperature diluted CTG (50 % PBS, 50 % CTG) was added on the cells. Plates were agitated on a plate agitator, covered by foil to protect from the light for 15 min and luminescence results were read by Tecan Infinite F200 PRO multimode microplate reader (according to the manufacturers recommendations).

### 2.7 RNA and DNA extraction

Total RNA and DNA extraction were performed the same way, by automated magnetic beads nucleic acid extraction with a King Fisher Flex (Thermo Fisher Scientific, Waltham, MA, USA), and the Nucleo Mag Vet Kit (Macherey-Nagel, Duren, Germany). RNA was extracted from 200 µl supernatants to assess EIAV viral release and DNA was extracted from cells, resuspended in 200 µl PBS, to assess EIAV vDNA and proviral integration. Supernatants and cells were frozen at −20 °C at determined timepoint (24, 72 or 144 h post-wash) before nucleic acid extraction.

### 2.8 Real time quantitative PCR

EIAV vDNA or vRNA was detected thanks to quantitative PCR (qPCR) and RT-qPCR using the late RT products primers (targeting *gag* gene) (supplementary material table S2) described previously (Jin, Chen, and Montelaro 2005) and Quantifast Sybr Green kit (Qiagen, Valencia, CA, USA), according to the manufacturer instructions. All RTqPCR and qPCR were performed on a Roche Light Cycler 480 thermocycler (Roche Diagnostics, Rotkreuz, Switzerland).

Relative viral RNA released in the culture supernatant quantities were determined using the 2^-DCt^ method (Cp_ref - Cp_treated), as a relative quantification of the Cp value of the viral release in the supernatant post-wash with a treatment (anti-HIV compound). For relative proviral integration quantification, the 2^-DDCt^ method was used ((Cp_ref_EIAV - Cp_ref_β-actin) - (Cp_treated_EIAV - Cp_treated_β-actin), using β-actin housekeeping gene primers (table S2) as a calibrator.

### 2.9 Statistical analysis

Statistical significance was assessed on R.v4.2.3. Pairwise paired *t*-test was performed with a BH correction. We present p.adj-values, differences were considered significant if p.adj<0.05 (*), p.adj<0.01 (**), p.adj<0.001 (***) or p.adj<0.0001 (****). All statistical results can be found in supplementary spreadsheet S1.

## 3 Results

### 3.1 Infection model of equine cells with EIAV

**Fig. 3:**
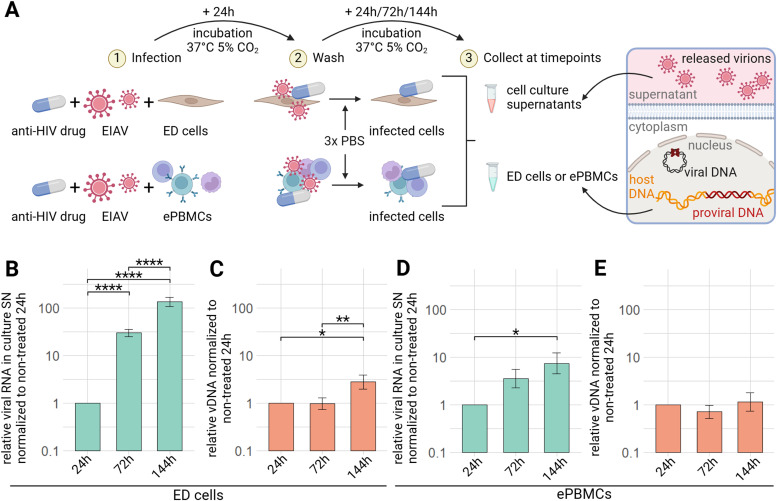
EIAV infection model of equine cells (A) Experimental design of *in vitro* EIAV infection of ED cells or equine PBMCs to quantify relative viral RNA (vRNA) in culture supernatants and relative viral DNA (vDNA) in cells; Relative quantification of EIAV vRNA in culture supernatants of infected ED cells (B) or ePBMCs (D), 24, 72 or 144 h post-wash, done after 24 h of infection, normalized by the 24 h timepoint, determined by RTqPCR. Relative quantification of EIAV vDNA in ED cells (C) or ePBMCs (E), 24, 72 or 144 h post-wash, done after 24 h of EIAV infection, normalized by β-actin Ct and 24 h timepoint determined by qPCR. Results are shown as mean ± sem, ED cells: *n* = 16 // ePBMCs: *n* = 6. * p.adj <0.05; ** p.adj <0.01; **** p.adj <0.0001 (as determined by pairwise paired Student's t-test, BH adjustment, see supplementary material spreadsheet S1 for all statistical analysis results) ED= equine dermal; ePBMCs= equine peripheral blood mononuclear cells; NT= non treated; SN: supernatant.

We are using an *in vitro* infection model of equine cells with EIAV that enables us to quantify the relative increase of viral RNA in culture supernatant and relative viral DNA (vDNA) of EIAV in the infected cells (comprising integrated proviral DNA and non-integrated vDNA) (Fig. 3A). In this study, we used equine dermal cells (ED cells), an equine fibroblast cell line, and equine peripheral blood mononuclear cells (ePBMCs).

In infected ED cells, an increase in viral release with time is observed. There is a hundred time increase of detected viral genome in the culture supernatant at 144 h compared to 24 h, and ten times more at 72 h, all significant (p.adj<0.0001) (Fig. 3B). Regarding vDNA in cells, there is a slight increase of relative vDNA quantity with time, which is significant from 24 to 144 h (p.adj<0.05) and from 72 to 144 h (p.adj<0.01) (Fig. 3C).

We get similar tendency with ePBMCs. We can observe an increase with time of detected viral genome in culture supernatants, significant between 24 and 144 h (p.adj<0.05) (Fig. 3D) and a plateau of relative vDNA quantity (Fig. 2E). These are primary cells with a lot of variability, the results were less consistent from one experiment to the other.

### 3.2 Anti-HIV compounds concentration range and cytotoxicity assessment

Before testing the antiviral effect of the selected compounds, we assessed their toxicity on the equine cells used in this study. We performed a cytotoxicity assay using 7 to 10 different compound concentrations on ED cells (supplementary material, table S1). Readouts were performed after 1, 24, 72 and 144 h. Cytotoxicity results on ED cells are shown as a heatmap (supplementary material, figure S1 and supplementary material spreadsheet S2) as a percentage of living cells. We can observe that most of the compounds are non-cytotoxic. This is the case for emtricitabine, lamivudine, zidovudine, enfuvirtide, fosamprenavir, raltegravir and bictegravir. For other compounds, we can see a cytotoxicity in the model over time. There is a slight observed cell death (<20 %) for nevirapine at 72 and 144 h of incubation for the highest concentration at 300µM. For abacavir, from 72 h highest concentrations (200 µM and 300 µM) result in a 50 % cell death, and for tenofovir we can observe a dose dependant toxicity starting from the 24 h of incubation, with a 40 % mortality for C10 (150 µM) at 24 h of incubation, to 50 % mortality at 144 h. Finally, regarding toxicity over time, we have serious cell mortality at highest concentrations (C08, C09 and C10) for atazanavir (80 µM, 100 µM, 124 µM), saquinavir (30µM, 50 µM, 65 µM) and tipranavir (100 µM, 140 µM, 166 µM), reaching 50 to 60 % mortality at C08 after 144 h of incubation, and over 90 % mortality at C10 for the same timepoint. Lastly, we can observe a loss of toxicity with time for some compounds like efavirenz or etravirine, that could be due to degradation.

Compound concentrations used to assess antiviral effects were chosen thanks to these results: minimum concentration tested (low), then a medium concentration, and a maximum concentration, (high) chosen to match a threshold of over 70 % of cell survival observed in this model (supplementary material table S1).

We assessed the cytotoxicity of the medium tested concentration on ED cells, on ePBMCs, as well as the combination of compounds on ED cells and the tested conditions were non cytotoxic. The cytotoxicity assay onED cells, ePBMCs and combination treatment on ED cells normalized results, as a percentage of living cells are in supplementary material spreadsheet S2.

### 3.3 Antiviral assessment of anti-HIV compounds against EIAV *in vitro* in ED cells

#### 3.3.1 Fusion inhibitor (FI)

**Fig. 4:**
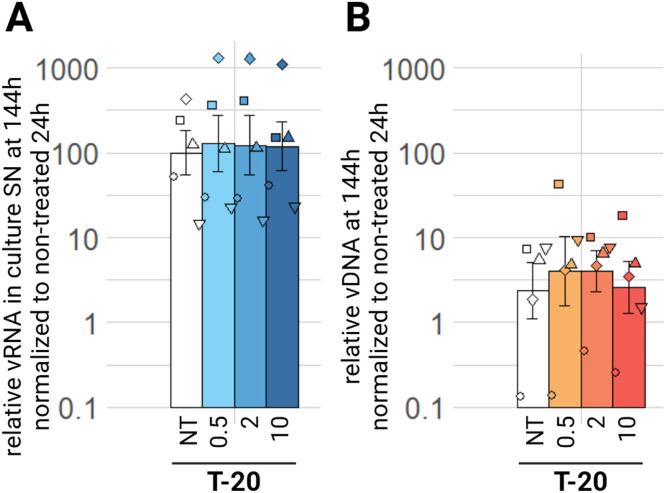
Effect of FI on EIAV infection in ED cells**.** ED cells were infected for 24 h with EIAV in presence of different T-20 concentrations (0.5 µM, 2 µM, 10 µM) then washed and EIAV infection was assessed by RTqPCR 144 h post-wash as relative vRNA in culture supernatants compared to a non-treated condition, all normalized by the 24 h non-treated condition (A) and by qPCR for relative EIAV vDNA in infected ED cells compared to a non-treated condition, all normalized by β-actin as a calibrator and by the 24 h non-treated timepoint (B). Results are shown as mean ± sem, *n* = 5 (Pairwise paired Student's t-test, BH correction was performed, see supplementary material spreadsheet S1 for all statistical analysis results) NT: non-treated; SN: supernatant; T-20: enfuvirtide; vDNA: viral DNA; vRNA: viral RNA.

We evaluated the efficacy of enfuvirtide (T-20), a fusion inhibitor. We observe no significant effect of any of the tested concentrations on the EIAV relative viral RNA in the culture supernatant or vDNA at 144 h (Fig. 4A, B), or at any timepoint (supplementary material figure S2 A, B, C, D). Enfuvirtide has no antiviral effect on EIAV infection of ED cells *in vitro*.

#### 3.3.2 Nucleoside reverse transcriptase inhibitors (NRTIs)

**Fig. 5:**
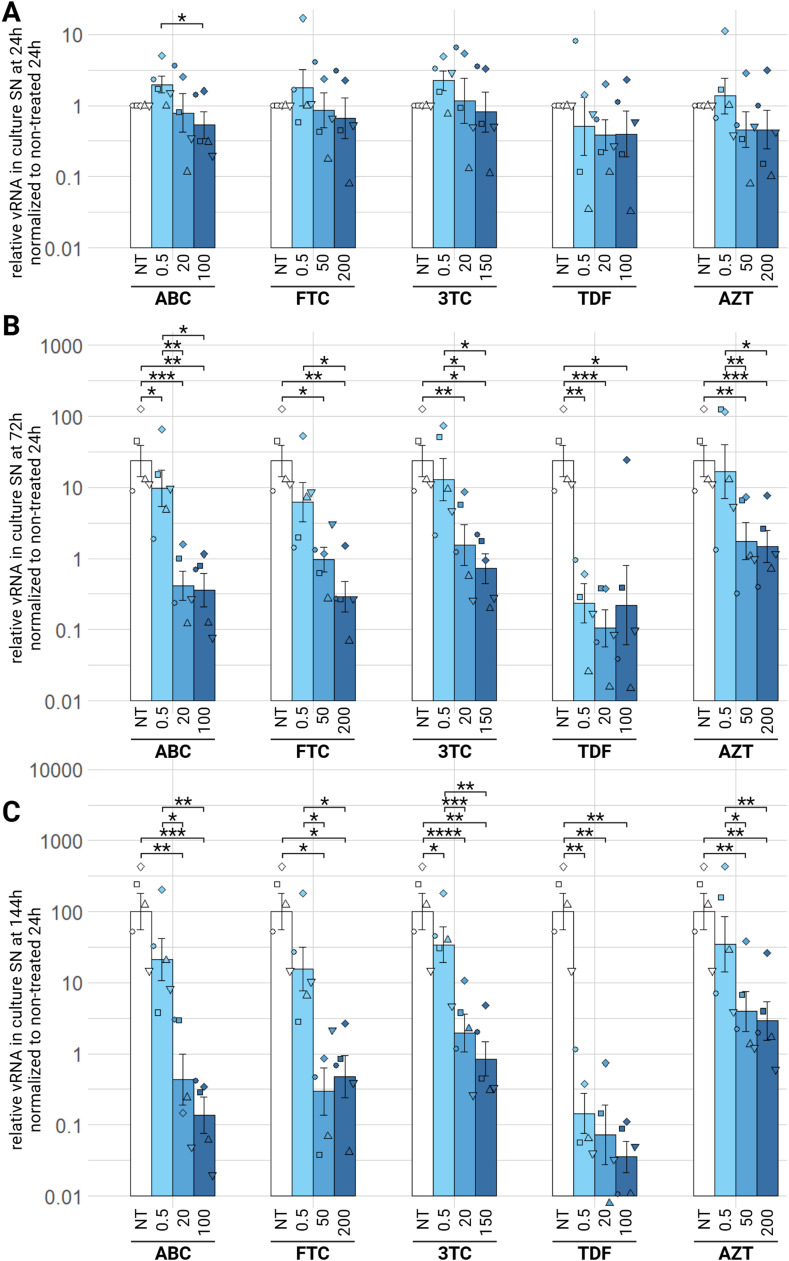
Effect of NRTI on EIAV infection in ED cells. ED cells were infected for 24 h with EIAV in presence of different NRTI compounds concentrations then washed and EIAV infection was assessed by RTqPCR 24 (A), 72 (B) or 144 (C) hours post-wash as relative vRNA in culture supernatants compared to a non-treated condition, all normalized by the 24 h non-treated condition. *p.adj <0.05; **p.adj <0.01; ***p.adj <0.001; **** p.adj <0.0001 (as determined by pairwise paired Student's t-test, BH correction, see supplementary material spreadsheet S1 for all statistical analysis results) ABC: abacavir; AZT: zidovudine; FTC: emtricitabine; NT: non-treated; TDF: tenofovir; vRNA: viral RNA; 3TC: lamivudine.

Abacavir (ABC), emtricitabine (FTC), lamivudine (3TC), tenofovir (TDF) and zidovudine (AZT), were tested for antiviral activity. After 24 h, we see no significant difference between control condition and treated conditions (Fig. 5A). At 72 h, there is still no difference, except for ABC 0.5 µM (padj<0.05) and TDF 0.5 µM (padj<0.01). there is a one log_10_ decrease of relative viral RNA between non treated condition and 20 µM or 100 µM of TDF (padj<0.001 or <0.05) and a half log_10_ decrease for remaining NRTIs tested at medium and high concentration (Fig. 5B). It is at 144 h that we observe the greatest difference between non-treated condition and treated conditions. Low concentration (0.5 µM) of 3TC (padj<0.05) and TDF (padj<0.01) show significant decrease of EIAV relative RNA in the culture supernatants. With medium and high concentrations, we observe a replication defect from a hundred to a thousand time for all 5 NRTIs compounds, with AZT having the least action (a hundred time decrease, at the highest concentration, 200 µM) and TDF having the strongest antiviral activity at the highest concentration (100 µM) with over thousand time decrease of viral release in the culture supernatants 1 at 144 h (Fig. 5C). There is no significant difference from medium to high compound concentration at all three tested timepoints, indicating that we reached the plateau of activity with the medium concentration.

**Fig. 6:**
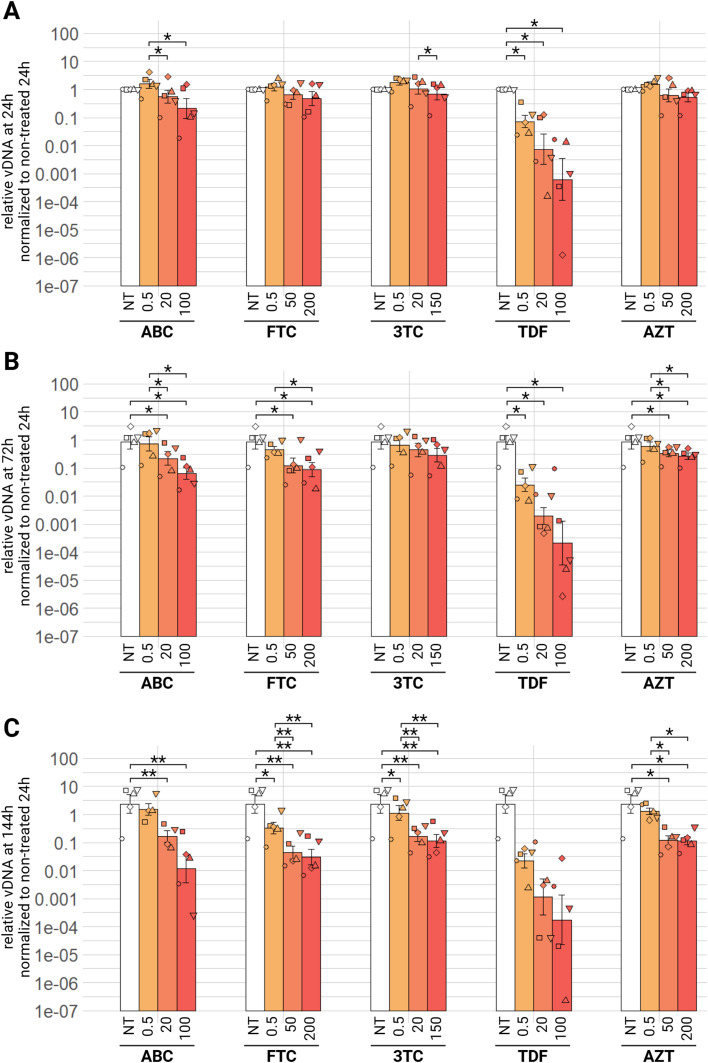
Effect of NRTI on EIAV infection in ED cells. ED cells were infected for 24 h with EIAV in presence of different NRTI compounds concentrations then washed and EIAV infection was assessed by qPCR 24 (A), 72 (B) or 144 (C) hours post-wash as relative vDNA in infected cells compared to a non-treated condition, all normalized by β-actin Ct and by the 24 h non-treated condition. Results are shown as mean ± sem, *n* = 5, *p.adj <0.05; **p.adj <0.01(as determined by pairwise paired Student's t-test, BH correction, see supplementary material spreadsheet S1 for all statistical analysis results) ABC: abacavir; AZT: zidovudine; FTC: emtricitabine; NT: non-treated; TDF: tenofovir; vRNA: viral RNA; 3TC: lamivudine.

Regarding the impact of NRTIs on EIAV total viral DNA in ED cells, we observe similar tendencies. Indeed, at 24 h, no compound has a significant effect on the decrease of EIAV vDNA, except for all concentrations of TDF, which significantly decrease the quantified relative EIAV vDNA ten times at 0.5 µM, a hundred time at 20 µM and finally a thousand time 100 µM, the highest tested concentration (padj<0.05) (Fig. 6A). At 72 h, ABC, FTC, TDF and AZT decrease significantly relative vDNA at medium and high tested concentrations, with TDF having the strongest impact at 100 µM, with a decrease of almost four log_10_ (padj<0.05) (Fig. 6B). 3TC has no significant effect at this timepoint, at any tested concentration. Finally, at 144 h, ABC and AZT decrease relative vDNA with medium and high concentration, but the lowest tested concentration (0.5 µM) has no significant effect (Fig. 6C). FTC and 3TC are effective at all three tested concentrations, with medium (50 µM and 20 µM respectively) and high (200 µM and 150 µM respectively) concentration being more effective than low concentration (0.5 µM), decreasing ten times the relative vDNA (padj<0.01). Regarding TDF we clearly see a tendency of an effect on vDNA, in a dose dependant manner: low concentration decreasing hundred times, medium concentration a thousand time and high concentration almost ten thousand times (padj<0.051). In summary, all five NRTIs tested against EIAV have an antiviral effect, observed as a decrease of relative viral RNA in culture supernatants or viral DNA in the cells, TDF having the strongest antiviral activity, and AZT the least in this *in vitro* model.

#### 3.3.3 Non-nucleoside reverse transcriptase inhibitors (NNRTIs)

**Fig. 7:**
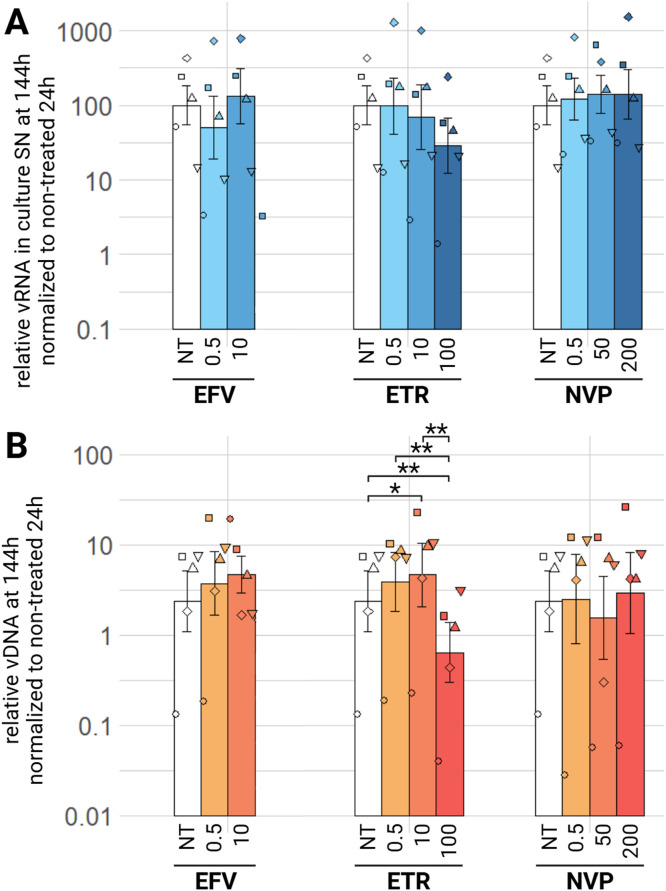
Effect of NNRTIs on EIAV infection in ED cells. ED cells were infected for 24 h with EIAV in presence of different NNRTIs compounds concentrations then washed and EIAV infection was assessed by RTqPCR 144 h post-wash as relative vRNA in culture supernatants compared to a non-treated condition, all normalized by the 24 h non-treated condition (A) and by qPCR for relative EIAV vDNA in infected ED cells compared to a non-treated condition, all normalized by β-actin as a calibrator and by the 24 h non-treated timepoint (B). Results are shown as mean ± sem, *n* = 5, *p.adj <0.05; **p.adj <0.01 (as determined by pairwise paired Student's t-test, BH correction was performed, see supplementary material spreadsheet S1 for all statistical analysis results)EFV: efavirenz; ETR: etravirine; NT: non-treated; NVP: nevirapine; SN: supernatant; vDNA: viral DNA; vRNA: viral RNA.

For the NNRTIs class, we tested efavirenz (EFV), etravirine (ETR) and nevirapine (NVP). For EFV, we only tested a low (0.5 µM) and medium concentration (10µM) as the compound appeared very toxic on ED cells at early timepoints. At any tested concentration, no significant decrease of the relative viral RNA in culture supernatants was observed at 144 h (Fig. 7A). Regarding relative EIAV vDNA, we observe a slight significant decrease at 144 h with 100 µM ETR concentration (Fig. 7B), but no significant change for EFV or NVP. For earlier timepoints, 24 and 72 h post-wash, the treated conditions have the same level of quantified viral RNA in the culture supernatant or relative vDNA as the non-treated condition (supplementary material S3 A, B, C, D). Efavirenz, etravirine and nevirapine have no antiviral effect against EIAV at any tested concentration, except for a slight decrease of vDNA with ETR that does not impact the relative viral RNA in the culture supernatant.

#### 3.3.4 Protease inhibitors (PIs)

**Fig. 8:**
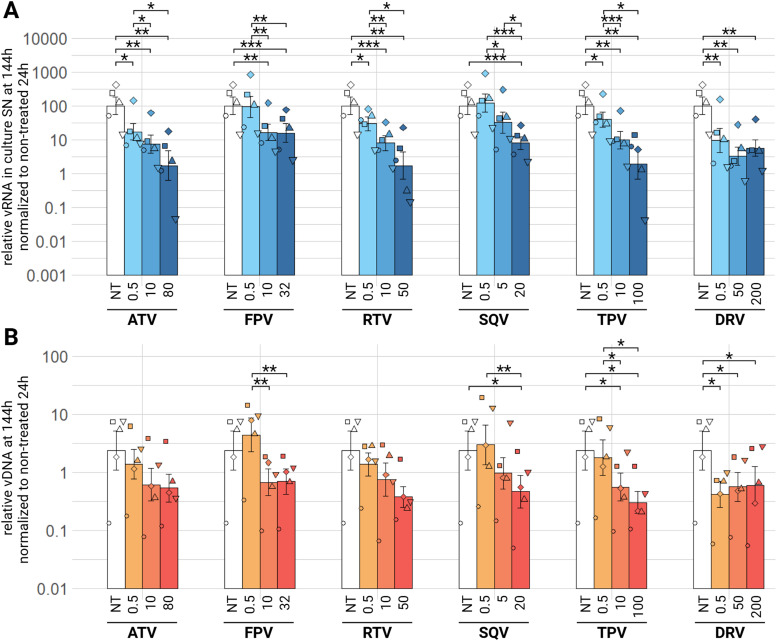
Effect of PIs on EIAV infection in ED cells. ED cells were infected for 24 h with EIAV in presence of different PIs compounds concentrations then washed and EIAV infection was assessed by RTqPCR 144 h post-wash as relative vRNA in culture supernatants compared to a non-treated condition, all normalized by the 24 h non-treated condition (A) and by qPCR for relative EIAV vDNA in infected ED cells compared to a non-treated condition, all normalized by β-actin as a calibrator and by the 24 h non-treated timepoint (B). Results are shown as mean ± sem, *n* = 5, *p.adj <0.05; **p.adj <0.01; **** p.adj <0.0001 (as determined by pairwise paired Student's t-test, BH correction, see supplementary material spreadsheet S1 for all statistical analysis results) ATV: atazanavir; DRV: darunavir; FPV: fosamprenavir; NT: non-treated; RTV: ritonavir; SN: supernatant; SQV: saquinavir; TPV: tipranavir; vDNA: viral DNA; vRNA: viral RNA.

For PIs drug class, we tested atazanavir (ATV), fosamprenavir (FPV), ritonavir (RTV), saquinavir (SQV), tipranavir (TPV) and darunavir (DRV). For medium and high tested concentrations of these 6 compounds, there is a significant 10 time decrease in viral load in the culture supernatants at 144 h (Fig. 8, A), the lowest concentration having a significant impact on relative viral RNA decrease for ATV, RTV, TPV (padj<0.05) and DRV (padj<0.01). Low concentration (0.5 µM) of FPV and SQV have no significant impact on relative viral RNA in culture supernatants. At 24 h no treatment has a significant impact on relative viral RNA in culture supernatants, but we observe the same tendencies at 72 h as at 144 h (supplementary material figure S4 A, B).

We observe a significant decrease in relative EIAV vDNA after treatment with PIs for medium and high concentrations of TPV, and for all concentrations of DRV at 144 h post-wash (Fig. 8, B). ATV, FPV and RTV show no significant decrease of relative vDNA with all three tested concentrations at 144 h post-wash (Fig. 8, B). No treatment with PIs has a significant effect on proviral integration at 72 h, same for 24 h, and surprisingly even a half log_10_ more relative vDNA with all treatments (supplementary material figure S4 C, D). ATV, FPV, RTV, SQV, TPV and DRV, all PIs have a significant effect on the decreasing of relative viral RNA in culture supernatants of EIAV infected cells, as well as a significant effect on vDNA, for FPV, SQV, TPV and DRV.

#### 3.3.5 Integrase strand transfer inhibitors (INSTIs)

**Fig. 9:**
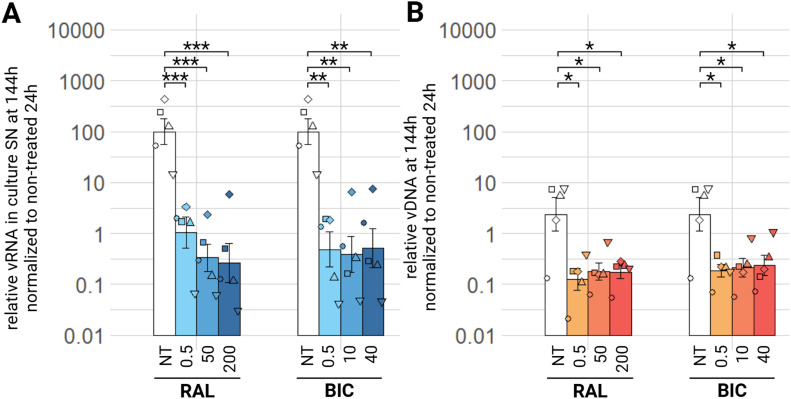
Effect of INSTIs on EIAV infection in ED cells. ED cells were infected for 24 h with EIAV in presence of different INSTIs compounds concentrations then washed and EIAV infection was assessed by RTqPCR 144 h post-wash as relative vRNA in culture supernatants compared to a non-treated condition, all normalized by the 24 h non-treated condition (A) and by qPCR for relative EIAV vDNA in infected ED cells compared to a non-treated condition, all normalized by β-actin as a calibrator and by the 24 h non-treated timepoint (B). Results are shown as mean ± sem, *n* = 5, *p.adj <0.05; **p.adj <0.01; **** p.adj <0.0001 (as determined by pairwise paired Student's t-test, BH correction, see supplementary material spreadsheet S1 for all statistical analysis results) BIC: bictegravir; NT: non-treated; RAL: raltegravir; SN: supernatant; vDNA: viral DNA; vRNA: viral RNA.

Raltegravir (RAL) and bictegravir (BIC) were tested as INSTIs. We can see at least a significant 100 time decrease of relative viral load in culture supernatants at 144 h with low concentration (0.5 µM) of RAL (padj<0.001), and two and half log_10_ decrease of relative viral RNA in culture supernatants with medium and high concentration (50 µM and 200 µM respectively) of RAL (padj<0.001), as well as with any tested concentration (0.5 µM, 10 µM or 40 µM) of BIC (padj<0.01) (Fig. 9, A). For both compounds, there is no dose-effect for the observed relative viral RNA in culture supernatants decrease in this *in vitro* model. At earlier timepoints, no difference is significant at 24 h (supplementary material figure S5, A), but for both compounds at all tested concentrations, there is a significant decrease in relative viral RNA in culture supernatants at 72 h (supplementary material figure S5, B). Regarding EIAV vDNA, both tested INSTIs show a significant 10 time decrease at any tested concentration 144 h (Fig. 9, B), but no significant change between treated conditions and control conditions regarding vDNA at 24 or 72 h (supplementary material figure S4 C, D).

Both RAL and BIC, from the INSTIs drug class decrease the relative EIAV viral RNA in culture supernatants and relative vDNA in infected cells, equally at any tested concentration and no compound showed greater effect than the other.

#### 3.3.6 Capsid inhibitor (CI)

**Fig. 10:**
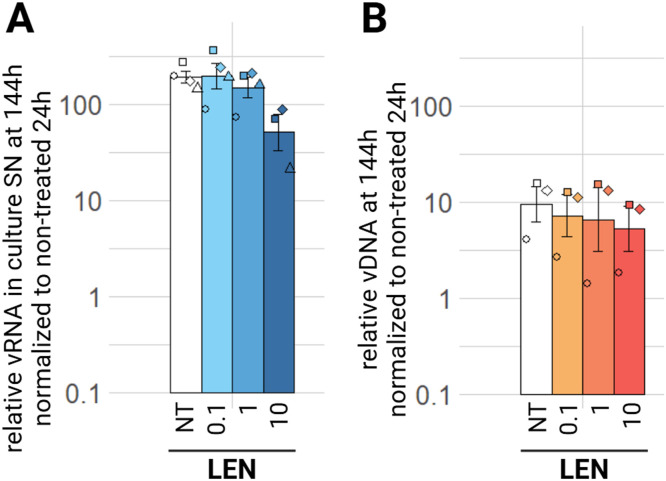
Effect of CI on EIAV infection in ED cells. ED cells were infected for 24 h with EIAV in presence of different LEN concentrations (0.1 µM, 1 µM, 10 µM) then washed and EIAV infection was assessed by RTqPCR 144 h post-wash as relative vRNA in culture supernatants compared to a non-treated condition, all normalized by the 24 h non-treated condition (A) and by qPCR for relative EIAV vDNA in infected ED cells compared to a non-treated condition, all normalized by β-actin as a calibrator and by the 24 h non-treated timepoint (B). Results are shown as mean ± sem, *n* = 5, (Pairwise paired Student's t-test, BH correction was performed, see supplementary material spreadsheet S1 for all statistical analysis results) LEN: lenacapavir; NT: non-treated; SN: supernatant; vDNA: viral DNA; vRNA: viral RNA.

Finally, we tested lenacapavir (LEN), the only capsid inhibitor yet approved to treat HIV-1. There is no significant decrease of relative viral RNA in the culture supernatants as well as in EIAV vDNA in the treated cells at 144 h (Fig. 10A, B). There is a non-significant tendency of decrease with the highest tested concentration (10µM), which can be observed with the relative viral release in supernatants at 144 h (Fig. 10, A) and even at 24 h (supplementary material, figure S6, A) significant this time at p.adj<0.05.

### 3.4 Antiviral assessment of anti-HIV compounds against EIAV *in vitro* in PBMC

**Fig. 11:**
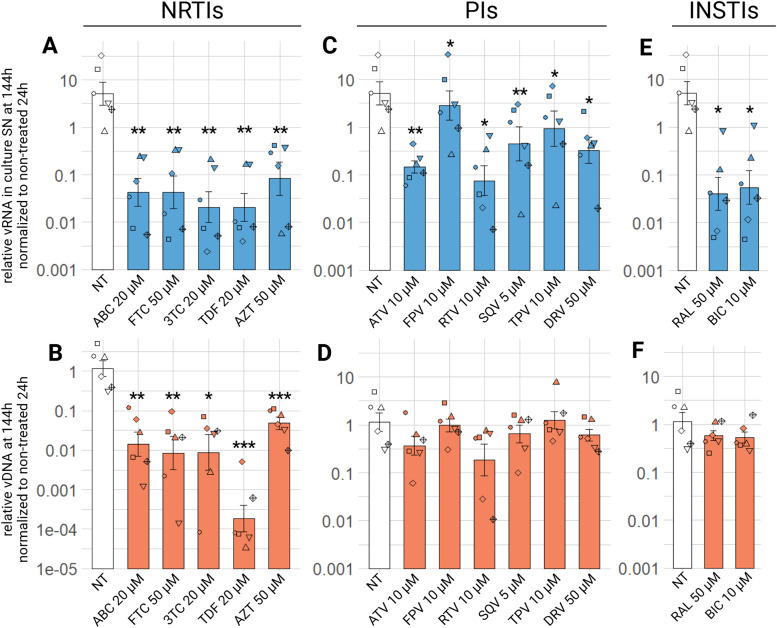
Effect of NRTIs, PIs and INSTIs on EIAV infection in ePBMCs. Equine PBMCs were infected for 24 h with EIAV in presence of different NRTIs, PIs or INSTIs compounds concentrations then washed and EIAV infection was assessed by RTqPCR 144 h post-wash as relative vRNA in culture supernatants compared to a non-treated condition, all normalized by the 24 h non-treated condition: NRTIs (A), PIs (C), INSTIs (E) and by qPCR for relative EIAV vDNA in infected ePBMCs compared to a non-treated condition, all normalized by β-actin as a calibrator and by the 24 h non-treated timepoint NRTIs (B), PIs (D) and INSTIs (F). Results are shown as mean ± sem, *n* = 6, *p.adj <0.05; **p.adj <0.01; *** p.adj <0.001 (determined by pairwise paired Student's t-test, BH correction, between control condition (NT) and treated condition, see supplementary material spreadsheet S1 for all statistical analysis results) ABC: abacavir; ATV: atazanavir; AZT: zidovudine; BIC: bictegravir; DRV: darunavir; FPV: fosamprenavir; FTC: emtricitabine; NT: non-treated;; RAL: raltegravir; RTV: ritonavir; SN: supernatant; SQV: saquinavir; TDF: tenofovir; TPV: tipranavir; vDNA: viral DNA; vRNA: viral RNA; 3TC: lamivudine.

Next, we aimed to confirm the observed antiviral effect observed on ED cells on ePBMCs. We tested the effect of NRTIs, PIs and INSTIs on EIAV infection of ePBMCs, at the medium tested concentration. There is a decrease of viral load in the supernatant at 144 h for all three drug classes. For NRTIs, there is over a one hundred times decrease with ABC, FTC, 3TC, TDF and AZT equally (Fig. 11A, padj<0.01)). For PIs a hundred times decrease of relative viral in culture supernatants can be observed with ATV (padj<0.01) and RTV (padj<0.05) (Fig. 11C). SQV and DRV decrease of ten times the relative viral RNA in culture supernatant (padj<0.01 and padj<0.05 respectively) (Fig. 11C). Finally, FPV and TPV have the least effect, but still a significant decrease of relative viral RNA in culture supernatants under these treatment conditions happens at the 144 h timepoint (padj<0.05) (Fig. 11C). Treatment with RAL or BIC, both tested INSTIs, results in a two log_10_ decrease of relative viral RNA in the culture supernatants (padj<0.05) (Fig. 11E). There is no significant decrease in viral load in supernatants with all three classes at 24 h (supplementary material S7 A, C, E), but at 72 h, we observe a significant decrease (p.adj<0.05) for all NRTIs, INSTIs and TPV (supplementary material S8, A, C, E). Regarding relative vDNA, we can observe a significant decrease in treated conditions compared to the non-treated condition with only NRTIs at 144 h (Fig. 11B). This is also verified for NRTIs at earlier timepoints: at 24 h post-wash (supplementary material figure S7, B) and 72 h (supplementary material figure S8, B). At all three timepoints, there is no significant decrease in relative proviral integration with PIs or INSTIs treatmentsalthough we observe a consistent tendency of decrease (supplementary material S7, D, F and S8, D, F).

### 3.5 Antiviral assessment of combination of anti-HIV compounds against EIAV *in vitro* in ED cells

Finally, we tested the combination of several effective compounds combined together, on ED cells. Bictegravir-emtricitabine-tenofovir combines two drug classes, one INSTI (BIC) and two NRTIs (FTC and TDF). Darunavir-emtricitabine-tenofovir combination also combines two drug classes, except that DRV is a PI.

**Fig. 12:**
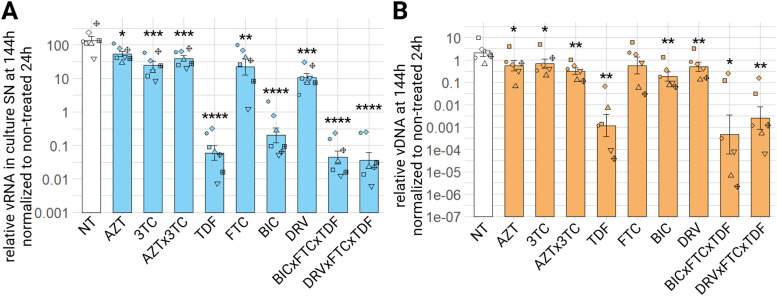
Effect of combination treatment on EIAV infection in ED cells. ED cells were infected for 24 h with EIAV in presence of different combination of antiretroviral compounds, then washed and EIAV infection was assessed by RTqPCR 144 h post-wash as relative vRNA in culture supernatants compared to a non-treated condition, all normalized by the 24 h non-treated condition (A) and by qPCR for relative EIAV vDNA in infected ED cells compared to a non-treated condition, all normalized by β-actin as a calibrator and by the 24 h non-treated timepoint (B). Results are shown as mean ± sem, *n* = 6, *p.adj <0.05; **p.adj <0.01; ***p.adj <0.001; **** p.adj <0.0001 (as determined by pairwise paired Student's t-test, BH correction, between control condition (NT) and treated condition, see supplementary material spreadsheet S1 for all statistical analysis results) AZT: zidovudine; AZTx3TC: zidovudine and lamivudine; BIC: bictegravir; BICxFTCxTDF: bictegravir and emtricitabine and tenofovir; DRV: darunavir; DRVxFTCxTDF: darunavir and emtricitabine and tenofovir; FTC: emtricitabine; NT: non-treated; TDF: tenofovir; SN: supernatant; vDNA: viral DNA; vRNA: viral RNA; 3TC: lamivudine.

Compounds were used at 0.5 µM in combination. In mono-treatment or combination, all compounds at 144 h have a significant effect on the decrease of relative viral RNA in culture supernatants. Indeed, for AZT, 3TC, combination AZT with 3TC (AZTx3TC) and FTC, we observe a significant half log_10_ decrease. For DRV alone, it's a one log_10_ decrease compared to the control condition. BIC alone reaches almost a thousand times decrease, and finally TDF, and the combination BIC with FTC and TDF (BICxFTCxTDF) and DRV with FTC and TDF (DRVxFTCxTDF) show over three log_10_ of decrease in viral RNA in culture supernatants compared to the control condition (Fig. 12A). We observe the same significant decrease tendency for relative vDNA in cells at the same timepoint of 144 h (Fig. 12B), with TDF and both combination of BICxFTCxTDF and DRVxFTCxTDF having the strongest impact on decreasing relative vDNA of EIAV in ED cells. Regarding relative viral RNA in culture supernatants, through all timepoints, the difference between treated conditions and non-treated conditions increases with time (supplementary material figure S9 A, C), whereas with vDNA the foldchange remains stable between non-treated and treated conditions (supplementary material figure S9 B, D).

The tested combinations of anti-HIV drugs in this *in vitro* infection model of ED cells with EIAV were shown to be efficient in reducing both vDNA and relative viral RNA in culture supernatants significantly. The sole combination of the NRTIs, zidovudine and lamivudine show a slight effect. There is no cumulative effect of the combination of NRTIs with an INSTI or a PIin both proviral integration and relative viral RNA in the culture supernatants.

## 4 Discussion

EIAV is a viral infection disseminated around the world. Unfortunately, as of today there is no therapeutic solution to avoid this infection and to limit its spreading. An attenuated vaccine was developed, but only authorized in China (Lin et al. 2011). To avoid the dissemination of the disease, some countries such as France, decide to euthanize the infected horses (Gaudaire et al. 2019). This policy is difficult to accept for the general population as well as for the horse owners, and some non-lethal solutions should be explored. In this work we present a possible therapeutic strategy focused on the use of antiretroviral compounds. HAART is now broadly used against HIV-1, a *lentivirus* like EIAV, and has constantly improved over the years with the development of new drug classes and the design of combination treatment, trying to avoid drug resistant variant and decreasing treatment burden for patients.

First, we assessed the cytotoxicity of the compounds on equine cells. We could observe a loss of toxicity for some of the compounds with time. Indeed, EFV and ETR showed a lesser toxicity over time (from 1 hour of incubation to 144 h), indicating that the compounds may be degraded. As this work was a targeted screening study, we chose not to add compounds at intermediate timepoints.

In this study, we identified thirteen anti-HIV-1 compounds that have a significant antiviral effect against EIAV *in vitro*, in equine cells. These thirteen compounds belong to three different antiretroviral drug classes, NRTIs, PIs and INSTIs. It was already reported that NRTIs have antiviral effect against FIV. Indeed, zidovudine, lamivudine, were investigated in several studies, showing an antiviral effect *in vitro* against FIV, in monotherapies as well as in NRTI combination treatment of abacavir with zidovudine and lamivudine, having synergistic effect in blocking *in vitro* replication of FIV (Bisset et al. 2002; Smyth et al. 1994; Schwartz et al. 2014). Furthermore, efficiency of zidovudine *in vivo* on FIV infected cats was also demonstrated (Hart and Nolte 1995). The INSTI raltegravir was reported as antiviral against FIV as well (Togami et al. 2013)**.**

In this *in vitro* study, all drugs within a specific drug class either exhibit or lack antiviral effects against EIAV, with varying degrees of effectiveness. We can hypothesize that other compounds from the NRTIs, PIs or INSTIs should have an antiviral effect against EIAV, and other compounds from NNRTIs would not, as long as they have the same molecular mechanism of action. It has already been described that NNRTIs, only inhibit HIV-1, not even HIV-2 or other tested lentiviruses such as SIV or FIV (Auwerx et al. 2002). This appears to be because NNRTIs are very specific to the HIV-1 RT protein. Regarding INSTIs, in the viral vDNA data, the decrease is only of one-fold log_10_, whereas the viral release is more impaired (three to four log_10_). Indeed, the viral cycle is blocked at the step with non-integrated viral DNA, that we do quantify, but there is little viral production because integration is impaired under this INSTI treatment. Interestingly in our *in vitro* assay, we observe an effect of tested PIs, like saquinavir, that decrease viral release, showing a significant antiviral effect against EIAV. PIs including saquinavir were previously described as ineffective against FIV and EIAV (Powell et al. 1996), but in another study, tipranavir, lopinavir and atazanavir were shown efficient against FIV *in vitro* (Norelli et al. 2008). An explanation could be that they have different amino acids in the binding site of inhibitors. Compounds that could inhibit different lentiviral proteases (HIV-1, FIV, EIAV) would be less likely to trigger drug resistant viral resistant, as it means that these compounds could bind different substrate binding pockets (Kervinen et al. 1998).

Differences and similarities between FIV protease and HIV-1 protease are well studied. The different viral proteases show preferences for their own substrates despite similar amino acid patterns (Schnölzer et al. 1996). This is an important piece of information for possible structure-based drug design of protease inhibitors against EIAV.

Regarding enfuvirtide, it is known that it binds to gp41 of HIV-1, it did not show any antiviral effect against EIAV, highlighting the functional structure difference between HIV-1 gp41 and EIAV gp45.

Finally, as monotherapy we tested the newly commercialized lenacapavir, inhibiting HIV-1 at two steps: impairing the uncoating of the capsid following the entry and impairing the reassembly of the capsid before the budding (Di Perri 2023). As expected with the differences in the capsid protein and the specificity of the molecule, our results show no specific activity against the EIAV capsid proteins.

We tested the published combination treatment of bictegravir-emtricitabine-tenofovir (Deeks 2018), which had a clear antiviral effect against EIAV *in vitro*. In monotherapy as well as in combination, TDF has a the strong antiviral effect, that could be linked to its possible half-life, longer than other's NRTIs (Holec et al. 2017). A combination treatment strategy is necessary, as antiretroviral drugs put a selection pressure and resistant virus variant can arise. Resistance to antiretroviral drugs was largely documented (Palmisano and Vella 2011; Larder, Darby, and Richman 1989; Borman, Paulous, and Clavel 1996; Vanangamudi et al. 2023) in the context of HIV-1 treatment.

Our study could provide a possible therapy to limit EIAV dissemination. Further *in vivo* studies are needed, but these antiviral compounds could be used as preventive measures, to control viremia of infected equids and avoid disease spread, while legal measures can be undertaken and improve life quality of EIAV infected equids.

## CRediT authorship contribution statement

**Cécile Schimmich:** Writing – review & editing, Writing – original draft, Visualization, Validation, Methodology, Investigation, Formal analysis, Data curation, Conceptualization. **Astrid Vabret:** Writing – review & editing, Validation, Supervision. **José-Carlos Valle-Casuso:** Writing – review & editing, Visualization, Validation, Supervision, Resources, Project administration, Methodology, Funding acquisition, Formal analysis, Data curation, Conceptualization.

## Declaration of competing interest

The authors declare that they have no known competing financial interests or personal relationships that could have appeared to influence the work reported in this paper.

## Data Availability

Data will be made available on request.

## Appendix Supplementary materials

Image, application 1

Image, application 2

Image, application 3

Image, application 4
