# Supplementary material for: Efficacy assessment of antiretroviral drugs against equine infectious anemia virus in vitro
Source: Virus Res. 2024 Dec 11;350:199503. doi: 10.1016/j.virusres.2024.199503 (PMC11699113; doi:10.1016/j.virusres.2024.199503)

**Figure S1:** Cytotoxicity of tested compounds on ED cells – Heatmap representation of cytotoxicity of anti-HIV compounds assessed on ED cells at 1, 24, 72 and 144 hours post-incubation at 37°C, 5% CO2, as a percentage of living cell. Results are shown as the mean percentage of living cells, normalized by the control condition of the experiment (C00, no compound) normalized to 100%. Experiment was done in three distinct biological replicates, all comprising at least 4 technical replicates for each condition.


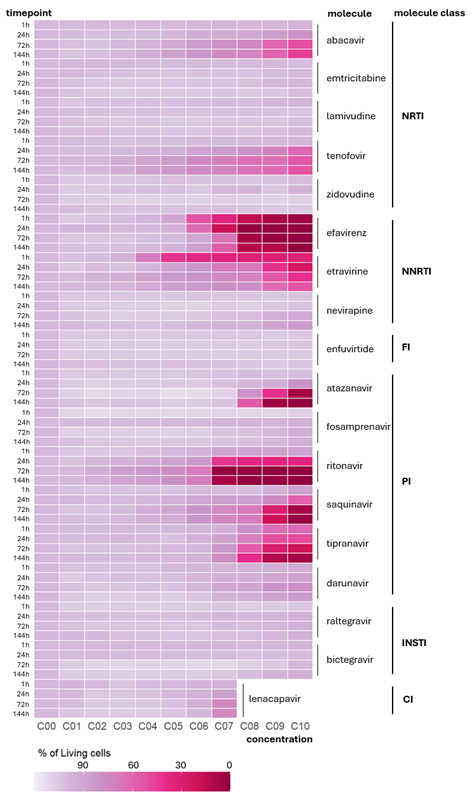


**Figure S2:** Effect of FI on EIAV infection in ED cells**.** ED cells were infected for 24 hours with EIAV in presence of different T-20 concentrations (0.5µM, 2µM, 10µM) then washed and EIAV infection was assessed by RTqPCR 24 hours (A) or 72 hours (B) post-wash as relative vRNA in culture supernatants compared to a non-treated condition, all normalized by the 24 hours non-treated condition and by qPCR for relative EIAV vDNA in infected ED cells compared to a non-treated condition, all normalized by β-actin as a calibrator and by the 24 hours non-treated timepoint 24 hours (C) or 72 hours (D) post-wash. Results are shown as mean +/- sem, n=5, *p.adj <0.05 (as determined by pairwise paired Student’s t test, BH correction was performed, see supplementary material spreadsheet S1 for all statistical analysis results)) NT: non-treated; SN: supernatant; T-20: enfuvirtide; vDNA: viral DNA; vRNA: viral RNA


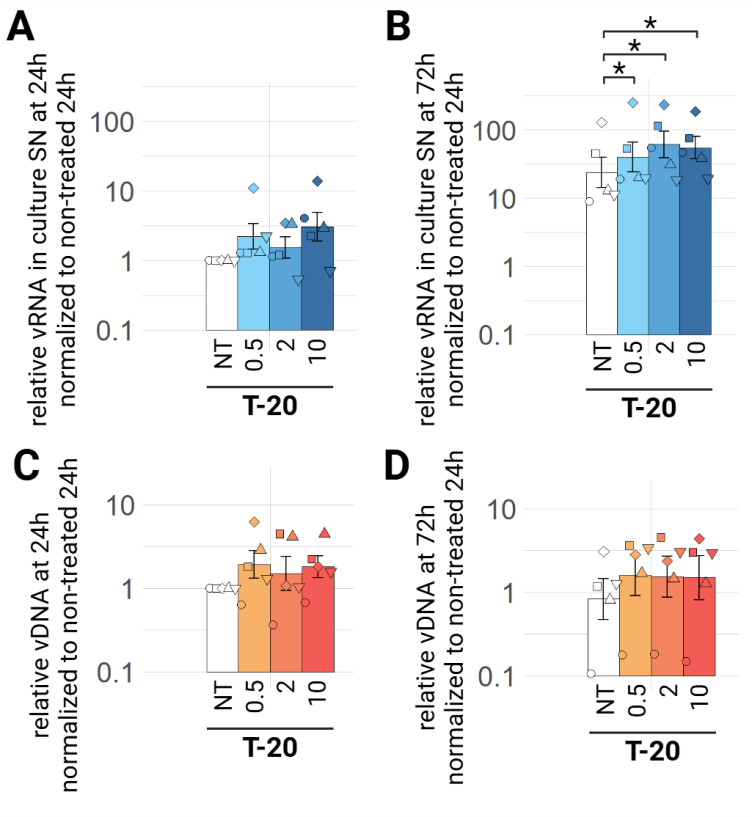


**Figure S3:** Effect of NNRTIs on EIAV infection in ED cells. ED cells were infected for 24 hours with EIAV in presence of different NNRTIs compounds concentrations then washed and EIAV infection was assessed by RTqPCR 24 hours (A) or 72 hours (B) post-wash as relative vRNA in culture supernatants compared to a non-treated condition, all normalized by the 24 hours non-treated condition (A) and by qPCR for relative EIAV vDNA in infected ED cells compared to a non-treated condition, all normalized by β-actin as a calibrator and by the 24 hours non-treated timepoint 24 hours (C) or 72 hours (D) post-wash. Results are shown as mean +/- sem, n=5, *p.adj <0.05 (as determined by pairwise paired Student’s t test, BH correction, see supplementary material spreadsheet S1 for all statistical analysis results)) EFV: efavirenz; ETR: etravirine; NT: non-treated; NVP: nevirapine; SN: supernatant; vDNA: viral DNA; vRNA: viral RNA


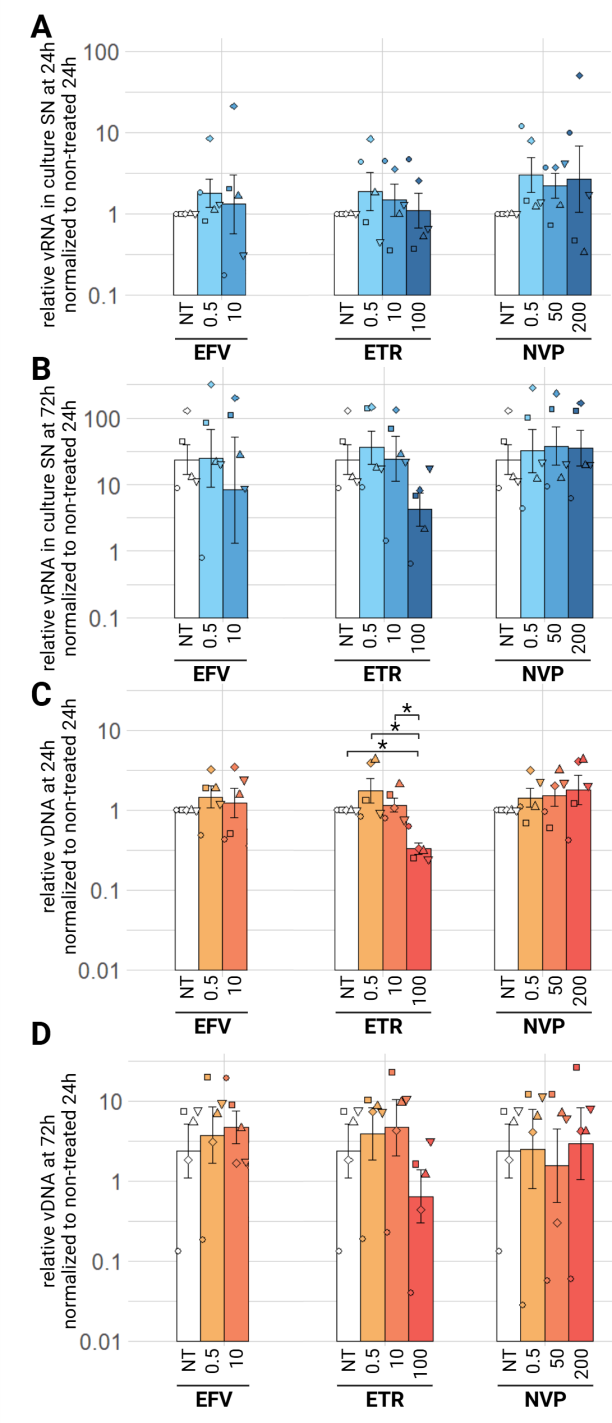


**Figure S4:** Effect of PIs on EIAV infection in ED cells. ED cells were infected for 24 hours with EIAV in presence of different PIs compounds concentrations then washed and EIAV infection was assessed by RTqPCR 24 hours (A) or 72 hours (B) post-wash as relative vRNA in culture supernatants compared to a non-treated condition, all normalized by the 24 hours non-treated condition and by qPCR for relative EIAV vDNA in infected ED cells compared to a non-treated condition, all normalized by β-actin as a calibrator and by the 24 hours non-treated timepoint 24 hours (C) or 72 hours (D) post-wash. Results are shown as mean +/- sem, n=5, *p.adj <0.05; **p.adj <0,01; **** p.adj <0,0001 (as determined by pairwise paired Student’s t test, BH correction, see supplementary material spreadsheet S1 for all statistical analysis results)) ATV: atazanavir; DRV: darunavir; FPV: fosamprenavir; NT: non-treated; RTV: ritonavir; SN: supernatant; SQV: saquinavir; TPV: tipranavir; vDNA: viral DNA; vRNA: viral RNA


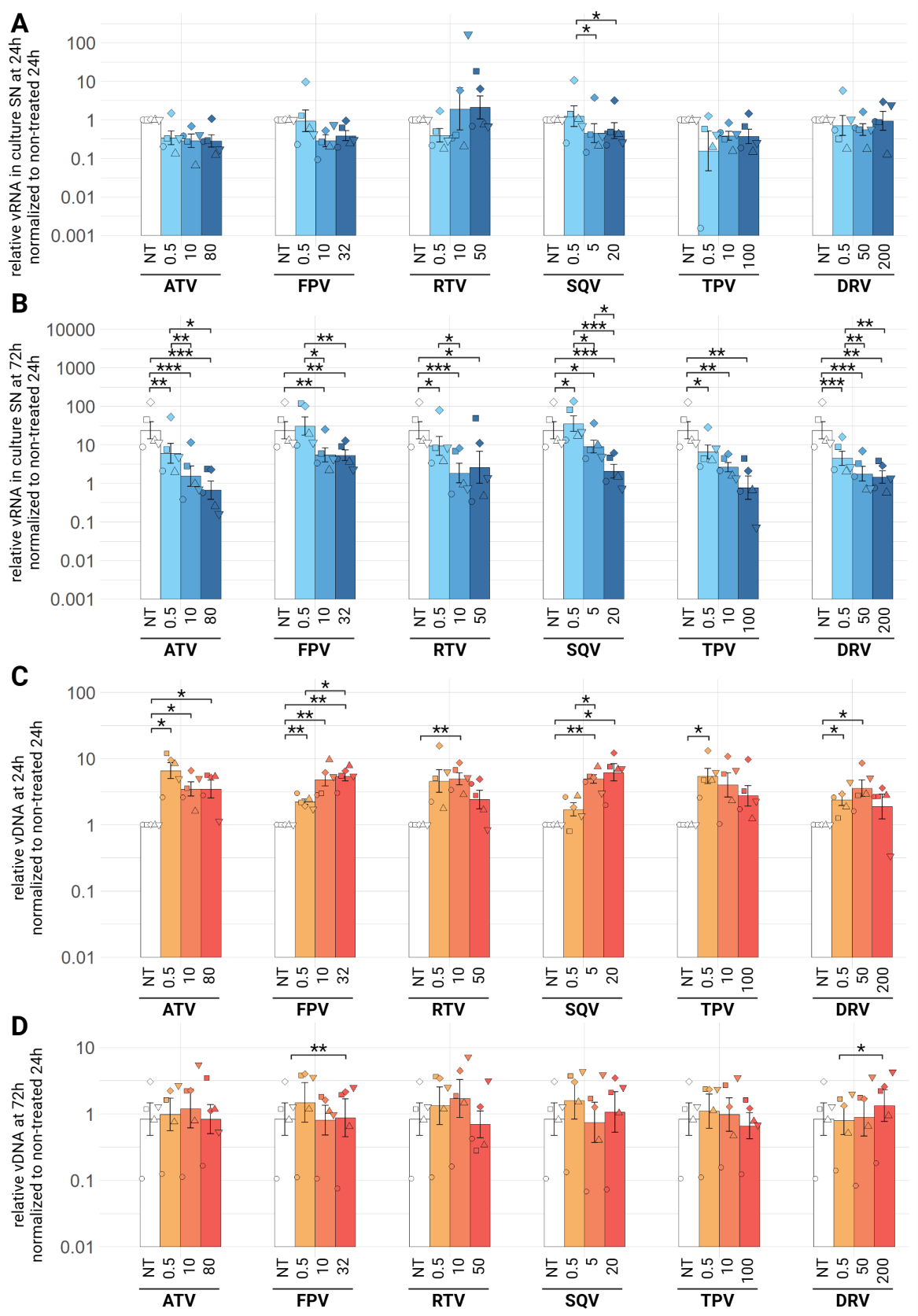


**Figure S5:** Effect of INSTIs on EIAV infection in ED cells. ED cells were infected for 24 hours with EIAV in presence of different INSTIs compounds concentrations then washed and EIAV infection was assessed by RTqPCR 24 hours (A) or 72 hours (B) post-wash as relative vRNA in culture supernatants compared to a non-treated condition, all normalized by the 24 hours non-treated condition (A) and by qPCR for relative EIAV vDNA in infected ED cells compared to a non-treated condition, all normalized by β-actin as a calibrator and by the 24 hours non-treated timepoint 24 hours (C) or 72 hours (D). Results are shown as mean +/- sem, n=5, *p.adj <0.05; **p.adj <0,01 (as determined by pairwise paired Student’s t test, BH correction, see supplementary material spreadsheet S1 for all statistical analysis results)) BIC: bictegravir; NT: non-treated; RAL: raltegravir; SN: supernatant; vDNA: viral DNA; vRNA: viral RNA


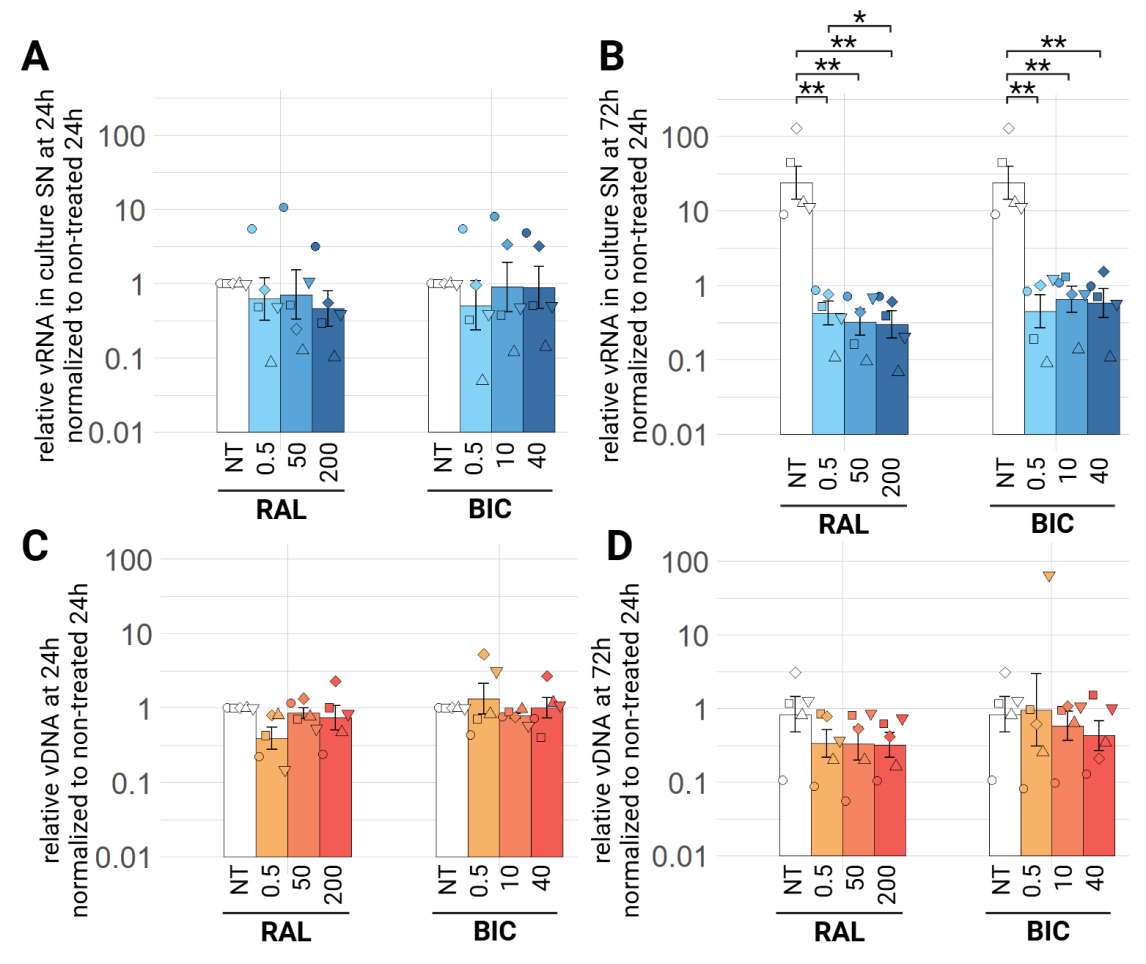


**Figure S6:** Effect of CI on EIAV infection in ED cells. ED cells were infected for 24 hours with EIAV in presence of different LEN concentrations (0.1µM, 1µM, 10µM) then washed and EIAV infection was assessed by RTqPCR 24 hours (A) or 72 hours (B) post-wash as relative vRNA in culture supernatants compared to a non-treated condition, all normalized by the 24 hours non-treated condition (A) and by qPCR for relative EIAV vDNA in infected ED cells compared to a non-treated condition, all normalized by β-actin as a calibrator and by the 24 hours non-treated timepoint 24 hours (C) or 72 hours post-wash. Results are shown as mean +/- sem, n=5, *p.adj <0.05 (as determined by pairwise paired Student’s t test, BH correction was performed, see supplementary material spreadsheet S1 for all statistical analysis results)) LEN: lenacapavir; NT: non-treated; SN: supernatant; vDNA: viral DNA; vRNA: viral RNA


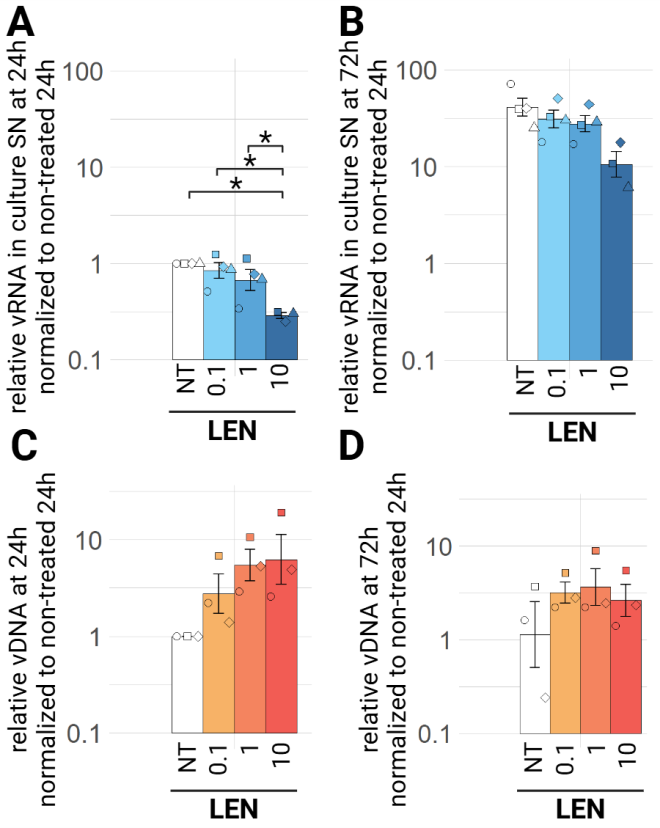


**Figure S7:** Effect of NRTIs, PIs and INSTIs on EIAV infection in ePBMCs 24 hours post-wash. Equine PBMCs were infected for 24 hours with EIAV in presence of different NRTIs, PIs or INSTIs compounds concentrations then washed and EIAV infection was assessed by RTqPCR 24 hours post-wash as relative vRNA in culture supernatants compared to a non-treated condition, all normalized by the 24 hours non-treated condition: NRTIs (A), PIs (C), INSTIs (E) and by qPCR for relative EIAV vDNA in infected ePBMCs compared to a non-treated condition, all normalized by β-actin as a calibrator and by the 24 hours non-treated timepoint NRTIs (B), PIs (D) and INSTIs (F). Results are shown as mean +/- sem, n=6, *p.adj <0.05; **p.adj <0,01; *** p.adj <0,001 (determined by pairwise paired Student’s t test, BH correction, between control condition (NT) and treated condition, see supplementary material spreadsheet S1 for all statistical analysis results)) ABC: abacavir; ATV: atazanavir; AZT: zidovudine; BIC: bictegravir; DRV: darunavir; FPV: fosamprenavir; FTC: emtricitabine; NT: non-treated; ; RAL: raltegravir; RTV: ritonavir; SN: supernatant; SQV: saquinavir; TDF: tenofovir; TPV: tipranavir; vDNA: viral DNA; vRNA: viral RNA; 3TC: lamivudine


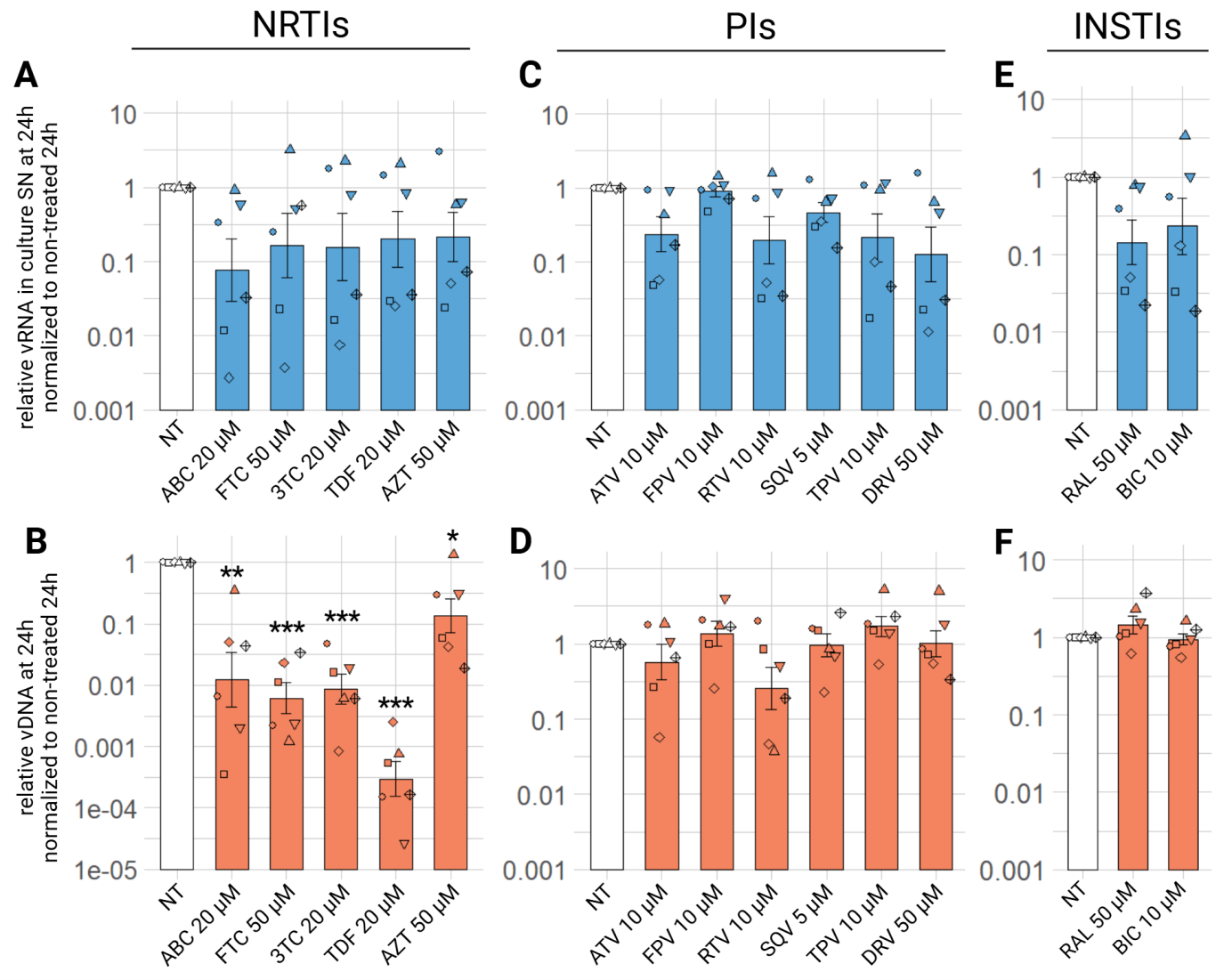


**Figure S8:** Effect of NRTIs, PIs and INSTIs on EIAV infection in ePBMCs 72 hours post-wash. Equine PBMCs were infected for 24 hours with EIAV in presence of different NRTIs, PIs or INSTIs compounds concentrations then washed and EIAV infection was assessed by RTqPCR 72 hours post-wash as relative vRNA in culture supernatants compared to a non-treated condition, all normalized by the 24 hours non-treated condition: NRTIs (A), PIs (C), INSTIs (E) and by qPCR for relative EIAV vDNA in infected ePBMCs compared to a non-treated condition, all normalized by β-actin as a calibrator and by the 24 hours non-treated timepoint NRTIs (B), PIs (D) and INSTIs (F). Results are shown as mean +/- sem, n=6, *p.adj <0.05; **p.adj <0,01 (determined by pairwise paired Student’s t test, BH correction, between control condition (NT) and treated condition, see supplementary material spreadsheet S1 for all statistical analysis results)) ABC: abacavir; ATV: atazanavir; AZT: zidovudine; BIC: bictegravir; DRV: darunavir; FPV: fosamprenavir; FTC: emtricitabine; NT: non-treated; ; RAL: raltegravir; RTV: ritonavir; SN: supernatant; SQV: saquinavir; TDF: tenofovir; TPV: tipranavir; vDNA: viral DNA; vRNA: viral RNA; 3TC: lamivudine


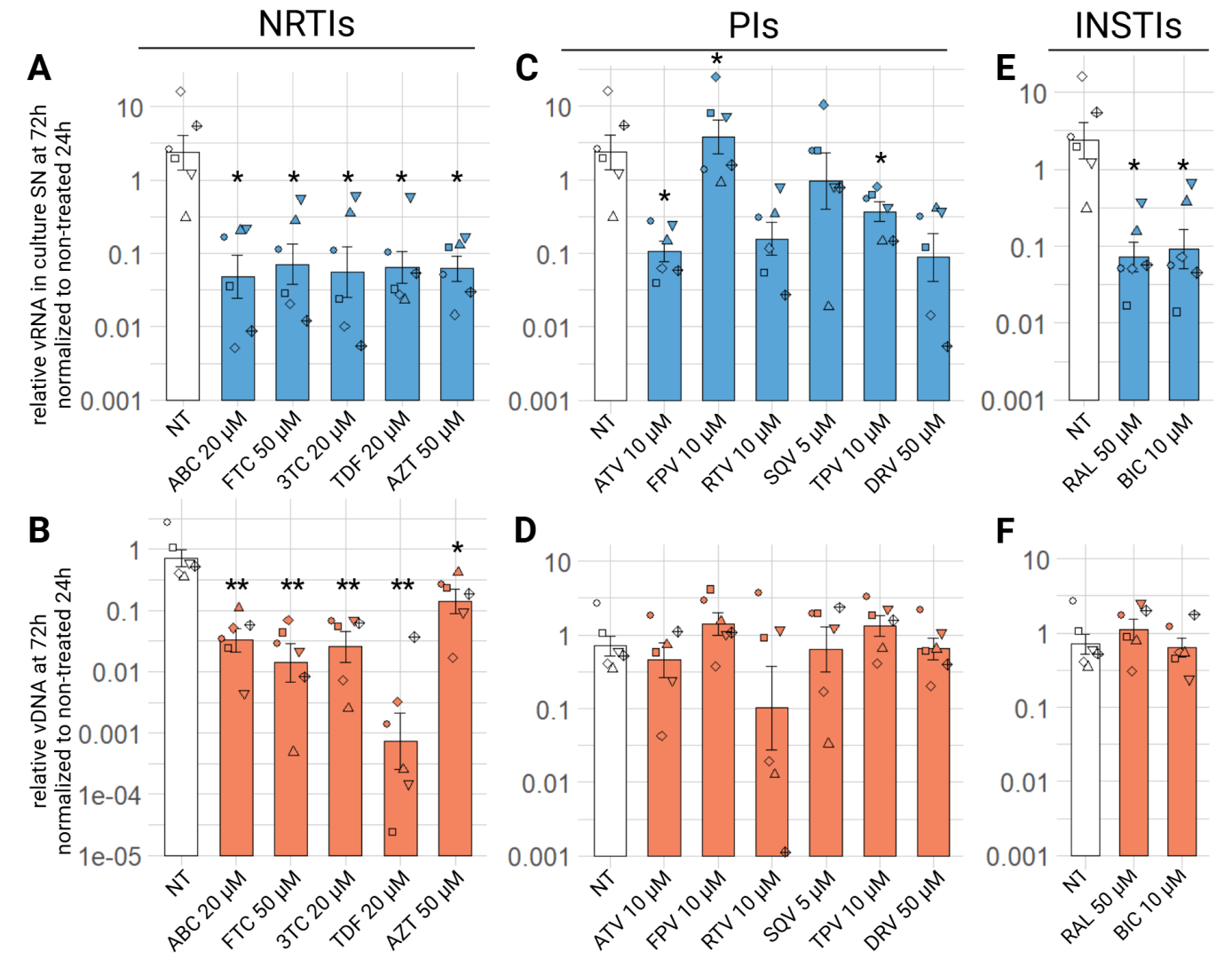


**Figure S9:** Effect of combination treatment on EIAV infection in ED cells. ED cells were infected for 24 hours with EIAV in presence of different combination of antiretroviral compounds, then washed and EIAV infection was assessed by RTqPCR 24 hours (A) or 72 hours (C) post-wash as relative vRNA in culture supernatants compared to a non-treated condition, all normalized by the 24 hours non-treated condition and by qPCR for relative EIAV vDNA in infected ED cells compared to a non-treated condition, all normalized by β-actin as a calibrator and by the 24 hours non-treated timepoint 24 hours (B) or 72 hours (D) post-wash. Results are shown as mean +/- sem, n=6, *p.adj <0.05; **p.adj <0.01; ***p.adj <0.001; **** p.adj <0.0001 (as determined by pairwise paired Student’s t test, BH correction, between control condition (NT) and treated condition, see supplementary material spreadsheet S1 for all statistical analysis results)) AZT: zidovudine; AZTx3TC: zidovudine and lamivudine; BIC: bictegravir; BICxFTCxTDF: bictegravir and emtricitabine and tenofovir; DRV: darunavir; DRVxFTCxTDF: darunavir and emtricitabine and tenofovir; FTC: emtricitabine; NT: non-treated; TDF: tenofovir; SN: supernatant; vDNA: viral DNA; vRNA: viral RNA; 3TC: lamivudine


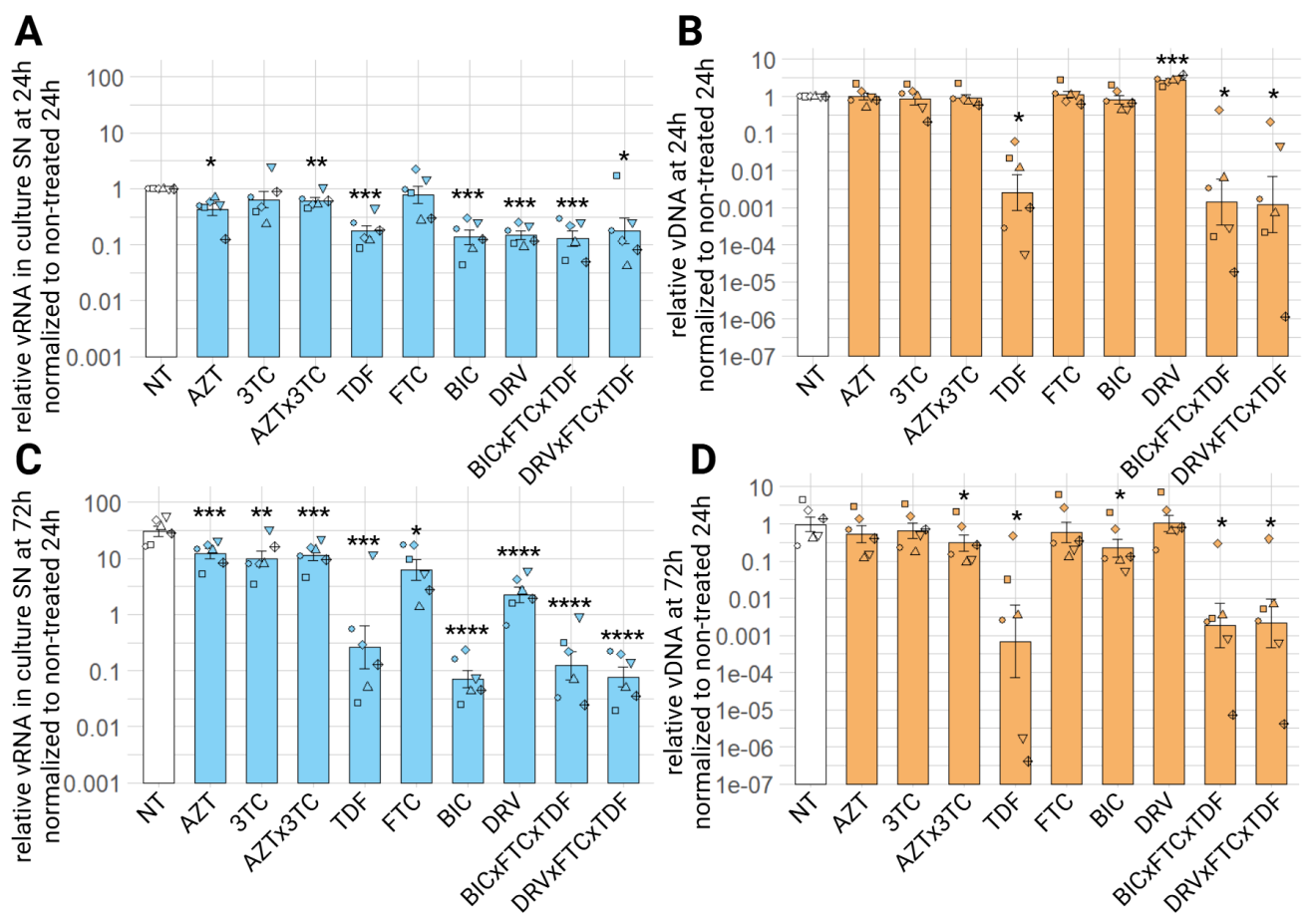

Supplement: Supplementary file 1 [file mmc1.docx]
