# Supplementary material for: Efficacy assessment of antiretroviral drugs against equine infectious anemia virus in vitro
Source: Virus Res. 2024 Dec 11;350:199503. doi: 10.1016/j.virusres.2024.199503 (PMC11699113; doi:10.1016/j.virusres.2024.199503)
Supplement: Supplementary file 2 [file mmc2.docx]

| **Class** | **Compound** | **C01** | **C02** | **C03** | **C04** | **C05** | **C06** | **C07** | **C08** | **C09** | **C10** | **Low** | **Medium** | **High** |
| --- | --- | --- | --- | --- | --- | --- | --- | --- | --- | --- | --- | --- | --- | --- |
| NRTIs | abacavir | 0.5 | 2 | 5 | 10 | 25 | 50 | 80 | 100 | 200 | 300 | 0.5 | 20 | 100 |
|  | emtricitabine | 0.5 | 2 | 5 | 10 | 25 | 50 | 80 | 100 | 200 | 300 | 0.5 | 50 | 200 |
|  | lamivudine | 0.5 | 2 | 5 | 10 | 20 | 40 | 50 | 75 | 100 | 150 | 0.5 | 20 | 150 |
|  | tenofovir | 0.5 | 2 | 5 | 10 | 20 | 40 | 50 | 75 | 100 | 150 | 0.5 | 20 | 100 |
|  | zidovudine | 0.5 | 2 | 5 | 10 | 25 | 50 | 80 | 100 | 200 | 300 | 0.5 | 50 | 200 |
| NNRTIs | efavirenz | 0.5 | 2 | 5 | 10 | 20 | 40 | 50 | 75 | 100 | 150 | 0.5 | 10 | NA |
|  | etravirine | 0.5 | 2 | 5 | 10 | 25 | 50 | 80 | 100 | 200 | 300 | 0.5 | 10 | 100 |
|  | nevirapine | 0.5 | 2 | 5 | 10 | 25 | 50 | 80 | 100 | 200 | 300 | 0.5 | 50 | 200 |
| FI | enfuvirtide | 3.10^-2^ | 6.10^-2^ | 0.125 | 0.25 | 0.5 | 1 | 2 | 5 | 8 | 11 | 0.5 | 2 | 10 |
| PIs | atazanavir | 0.125 | 0.25 | 0.5 | 2 | 10 | 20 | 50 | 80 | 100 | 124 | 0.5 | 10 | 80 |
|  | darunavir | 0.5 | 2 | 5 | 10 | 25 | 50 | 80 | 100 | 200 | 300 | 0.5 | 50 | 200 |
|  | fosamprenavir | 3.10^-2^ | 6.10^-2^ | 0.125 | 0.25 | 0.5 | 2 | 5 | 10 | 20 | 32 | 0.5 | 10 | 32 |
|  | ritonavir | 0.5 | 2 | 5 | 10 | 25 | 50 | 80 | 100 | 200 | 300 | 0.5 | 10 | 50 |
|  | saquinavir | 0.25 | 0.5 | 2 | 5 | 10 | 20 | 25 | 30 | 50 | 65 | 0.5 | 5 | 20 |
|  | tipranavir | 0.25 | 0.5 | 2 | 5 | 10 | 20 | 50 | 100 | 140 | 166 | 0.5 | 10 | 100 |
| INSTIs | raltegravir | 0.5 | 2 | 5 | 10 | 25 | 50 | 80 | 100 | 200 | 300 | 0.5 | 50 | 200 |
|  | bictegravir | 6.10^-2^ | 0.125 | 0.325 | 10 | 2 | 5 | 10 | 20 | 30 | 42 | 0.5 | 10 | 40 |
| CIs | lenacapavir | 1.25.10^-5^ | 2.5.10^-5^ | 5.10^-5^ | 1.10^-4^ | 1.10^-3^ | 1 | 10 | NA | NA | NA | 0.1 | 1 | 10 |

**Table S1:** Anti-HIV compounds used in this study and concentrations (µM) assessed in cytotoxicity assay (C01 to C10) and concentrations (µM) used for antiviral activity test (low, medium, high)

| **Primer name** | **Primer sequence 5’ to 3’** |
| --- | --- |
| late RT products-F | GGAGCCTTGAAAGGAGGGCCACTAAA |
| late RT products-R | TTGTTGTGCTGACTCTTCTGTTGTATCGGG |
| β-actin-F | CAGCACAATGAAGATCAAGATCATC |
| β-actin-R | CGGACTCATCGTACTCCTGCTT |

**Table S2:** Primer sequences used in this study – RT: reverse transcriptase
